# Chronic, Multi-Site Recordings Supported by Two Low-Cost, Stationary Probe Designs Optimized to Capture Either Single Unit or Local Field Potential Activity in Behaving Rats

**DOI:** 10.3389/fpsyt.2021.678103

**Published:** 2021-08-05

**Authors:** Miranda J. Francoeur, Tianzhi Tang, Leila Fakhraei, Xuanyu Wu, Sidharth Hulyalkar, Jessica Cramer, Nathalie Buscher, Dhakshin R. Ramanathan

**Affiliations:** ^1^Mental Health Service, VA San Diego Healthcare System, San Diego, CA, United States; ^2^Department of Psychiatry, University of California, San Diego, San Diego, CA, United States

**Keywords:** electrophysiology, single unit, field potentials, multi-site recordings, rodent models, fixed array

## Abstract

Rodent models of cognitive behavior have greatly contributed to our understanding of human neuropsychiatric disorders. However, to elucidate the neurobiological underpinnings of such disorders or impairments, animal models are more useful when paired with methods for measuring brain function in awake, behaving animals. Standard tools used for systems-neuroscience level investigations are not optimized for large-scale and high-throughput behavioral battery testing due to various factors including cost, time, poor longevity, and selective targeting limited to measuring only a few brain regions at a time. Here we describe two different “user-friendly” methods for building extracellular electrophysiological probes that can be used to measure either single units or local field potentials in rats performing cognitive tasks. Both probe designs leverage several readily available, yet affordable, commercial products to facilitate ease of production and offer maximum flexibility in terms of brain-target locations that can be scalable (32–64 channels) based on experimental needs. Our approach allows neural activity to be recorded simultaneously with behavior and compared between micro (single unit) and more macro (local field potentials) levels of brain activity in order to gain a better understanding of how local brain regions and their connected networks support cognitive functions in rats. We believe our novel probe designs make collecting electrophysiology data easier and will begin to fill the gap in knowledge between basic and clinical research.

## Introduction

Animal models with high translational validity to clinical data are critical for understanding the neurobiology of psychiatric disorders with the aim to develop novel therapeutic strategies. Rodents are useful to model complex aspects of cognition and decision-making which are impaired in addiction, neuropsychiatric and developmental disorders, or after brain injury (1–3). Such “translational” tasks include those that measure various aspects of attention and vigilance (4, 5), motivation and reward learning (6, 7), impulsivity (2, 8–11) executive function (1, 12) and even a concept as abstract as sunk costs (13). Translating such tasks across species has offered the ability to understand the effects of controlled genetic or environmental manipulations on behavior and cognition (14–19), and further to test how pharmacologic (7, 10, 20–22) or neuromodulatory (23, 24) therapies might remediate cognitive deficits.

Filling the gap in translational knowledge between clinical and animal research depends on common physiological circuits which underlie these cognitive behaviors across species. However, it is clear, for example, that prefrontal cortex in rats does not clearly mimic that in humans (25–28). Thus, ensuring at least some common brain circuitry (i.e., orbitofrontal involvement on the same task across species) is an important step in validating the translational utility of such tasks. In animal models in particular, electrophysiology recordings spanning across different temporal and spatial scales (i.e., single units and field potentials) is instrumental for comparing neural circuits across species (29–31) and to further investigate abnormal rhythmic or voltage patterns observed during disease states (3, 32). Single unit activity has long been the “gold-standard” of understanding how activity in particular brain regions is linked with behavior (33, 34). Local field potentials (LFPs) have increasingly been used as an intermediary measure of nervous system function that can be compared with both single unit activity and EEG/MEG (35). Importantly field potentials occur in rhythmic, oscillatory patterns, and capture information that is directly relevant to local spiking (32, 36–38). In addition, field potentials are used to characterize larger-scale brain interactions relevant to behavior (35, 39). Thus, single units and field potential activity offer complementary views of local neural activity occurring within brain regions.

There are many technical obstacles to this which have prevented widespread adoption of *in vivo* collection of brain activity in animal behavioral researchers. First, standard systems-neuroscience tools for probing *in vivo* neural function are costly and time-consuming and require specialized equipment, making large-scale studies (as are typically needed when assessing behavioral effects of genetic, environmental, or pharmacological manipulations) challenging to implement. Thus, developing tools and approaches for mapping brain activity in rodents that are cost-effective, scalable and affordable for labs looking to add electrophysiology as a secondary methodology is important. Second, many rodent models of environmental manipulations such as traumatic brain injury, stroke, or chronic stress, changes in neural activity and behavior evolves over the course of weeks to months. Thus, developing robust methods that allow for longitudinal data collection are critical. Finally, there are no simple and cost effective strategies of mapping brain activity across many areas simultaneously in rodents that are similar to whole-brain imaging methods in humans (fMRI, MEG, EEG). Such studies, paired with data-driven approaches, have identified important patterns of activity linked with cognition and psychiatric illness (identification of default-mode-network being perhaps the most striking example). Therefore, methods that can characterize activity in multiple brain regions in rats simultaneously, without prior knowledge of a target brain region, is advantageous.

To address these, we describe here two novel electrode designs for measuring either single unit or local field potentials, while prioritizing a low-cost and versatile design. Our 32–64 channel stationary single unit probes use a separate microwire design to capture high quality spiking activity within the anterior cingulate cortex and orbitofrontal cortex in rats performing a behavioral inhibition task stably over many weeks. Microwires are arranged in sets of fixed cannula bundles with recording tips spread in a fan-like array to minimize damage upon insertion (40–44). Importantly, our probes are around 1/10 the cost of similar channel density probes from commercial vendors (45–51). We also describe the fabrication of a customized LFP probe capable of measuring brain activity from up to 32 different brain locations across months of time using a similar low-cost, flexible design. The novelty of this approach is that the careful arrangement of our wires provides an unbiased measure of wide-spread brain activity across multiple networks with temporal precision. Both probes can measure brain activity over long time spans often needed for genetic/environmental manipulations. The rationale, fabrication, implantation, and quantifiable results are presented here. We believe these approaches will allow even non-physiologists the ability to measure brain activity to complement their behavioral investigations in rodents which is necessary to perpetuate our understanding of human neurological disorders.

## Materials and Equipment

### Probe Design Background

There are two main approaches to collecting chronic extracellular recordings in awake, behaving animals. With a stationary probe design, wires are lowered to the target brain depth during surgery and are not adjusted after implantation. Fixed devices can have up to 384 recording sites, targeting many brain areas simultaneously as is seen with the Neuropixels silicon probe (46–48). The other approach utilizes a microdrive to move electrodes along the vertical axis to advance after surgical implantation (52–58). Microdrives are advantageous for reaching neural dense areas and moving past the site of initial injury/ inflammatory response, however fabrication can be more challenging and expensive than fixed implants and are typically limited in the number of multi-site trajectories they can support (52, 53, 55–57). Favoring a simple, cost-effective design, we built a custom stationary probe with 32–64 electrodes for multi-site single unit recordings and another probe with 32 channels optimized to record “brain-wide” field potentials. Table 1 lists parts necessary to build our probes including supplier, stock number, and price estimate. The cost of our stationary probes, including the wires, gold pins, and electrode interface board (EIB), is ~$150 per probe (See Table 1 parts list and pricing). Note that our cost estimate does not include instruments for impedance adjustment, surgical apparatus, or recording equipment. **Table 3** compares our probe design and quantifiable results with other custom and commercial probes.

**Table 1:** Suggested materials for single unit and field potential probes.

**Product**	**Supplier**	**Stock number**	**Price**
Miniature stainless steel tubing	McMaster-Carr	8988K81	$7.01/ft
Nichrome microwires	Sandvick	PX000004	$750/1,000 ft
Super glue—Gel control	Loctite	1363589	$3.97 ea
Tungsten-carbide micro dissecting scissors	Stoelting	52132-29P	$205 ea
32 channel electrode interface board	Neuralynx	EIB-36-PTB	$135.00 ea
Small EIB pins for 32 CH EIB	Neuralynx	N/A	$175.00/case (1,000 pins)
64 channel electrode interface board	Open Ephys	N/A	€149 ea
Large EIB pins for 64 CH EIB	Open Ephys	OEPS-7012	€280/case (1,000 pins)
EIB accessories- serrated jaw pliers, precision tweezers and magnet	Open Ephys	OEPS-7020	€135/set
nanoZ adaptor	Neuralynx	ADPT-NZ-N2T-32	$200
nanoZ	Neuralynx	NZ-N2T-32	$2,200
Gold plating solution	Neuralynx	N/A	$200/10 mL
Tungsten 99.95% insulated wire	California fine wire company	M494860	$0.50/ piece
Dental cement kit	Stoelting	51459	$100 ea
C&B MetaBond	Parkell, Inc.	S380	$456.00/kit
Solder	Kester	24-6040-0027	$36.31/spool
Screws	Fine science tools	19010-00	$88/ case (100 screws)
PFA-coated stainless steel wire	A-M systems	790900	$145/spool

### Stationary Single Unit Probe Design

**Figure 1:**
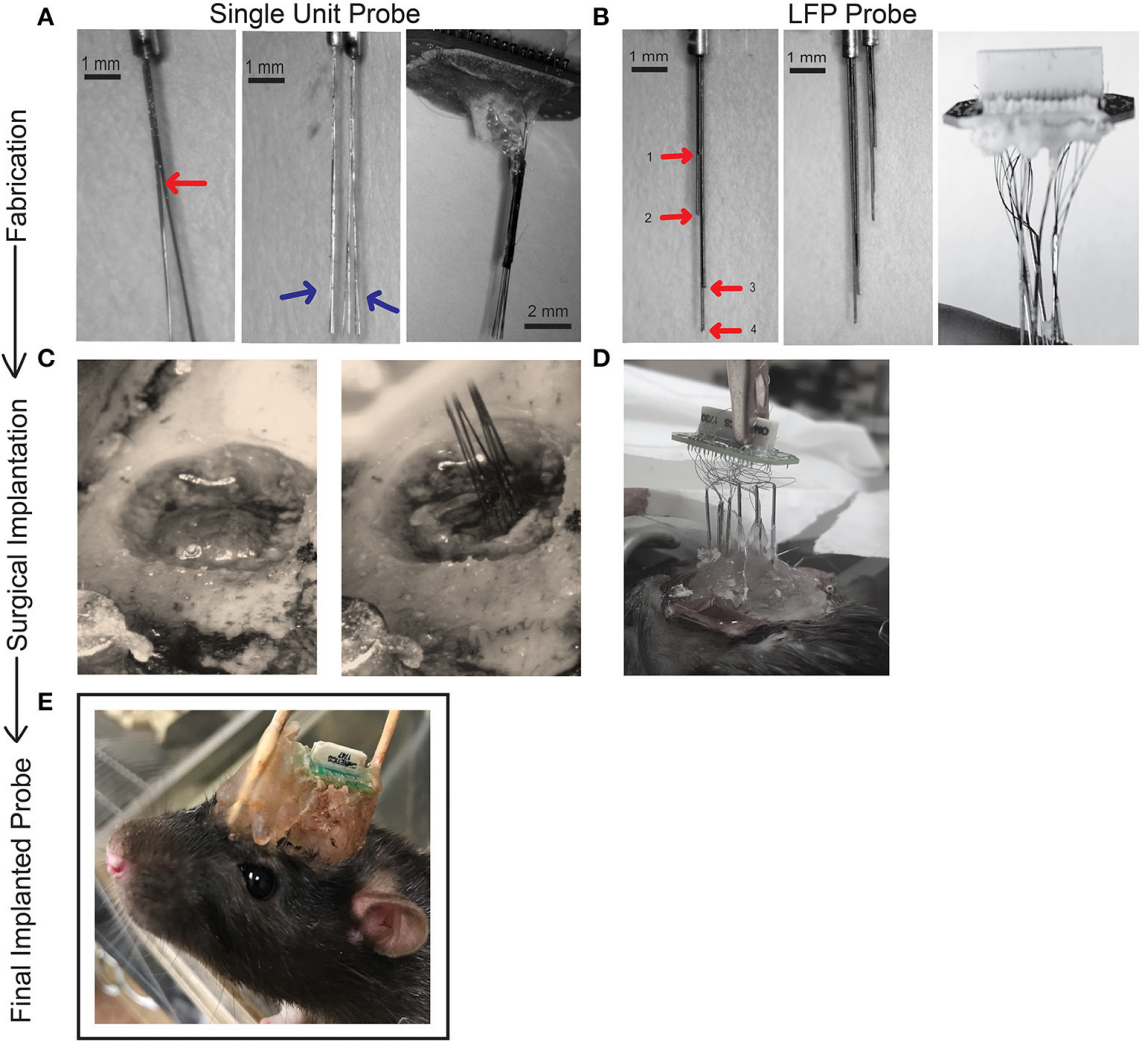
Schematic showing process of fabrication **(A,B)**, surgical implantation **(C,D)**, and final chronic fixed implant **(E)** for single unit and LFP probes. **(A)** Fabrication steps for one 32-channel single unit probe. Bifurcations of a bundle of eight wires (Sandvik). Wires are separated into bundles of four using superglue to secure and then further separated into two bundles of two wires. The red arrow points to the first separation and blue arrows to the second separation. The brush-like formation reduces potential damage to brain tissue upon insertion. Two of these cannulas (eight electrodes) were glued together in parallel (16 electrodes). A 32-electrode assembly is made by combining two, 16-electrode bundles. All 32 wires are then attached to an EIB board and secured with gold pins (Neuralynx) and covered for protection with dental cement and superglue. **(B)** To fabricate our LFP probe 32, 50 μm tungsten wires (California Fine Wire) were pre-cut to 10 cm in length and four wires inserted into a cannula (Mcmaster-Carr). The length of each wire extending beyond the cannula is measured according to the desired dorsal/ventral (DV) measurement of the target brain region. In our design, each wire in a cannula has a unique length, shown with red arrows and labeled 1–4. Two sets of cannulae are shown for comparison that will target different AP/ML locations. Eight sets of such electrodes are prepared (eight cannulas housing four wires) and similarly secured to the EIB board with gold pins and protected with a small layer of dental cement. **(C)** The craniotomy drilled for surgical implantation of the 32- channel single unit probe. Ground screw and anchor screws are secured with dental cement to the skull before electrodes are inserted. The implant is slowly lowered under stereotaxic control to the target depth. **(D)** We have found two successful methods for implanting our LFP probes (1) securing the cannula to the EIB board may occur before surgery (2) the cannula may be lowered one at a time to their target locations, secured, and then attached to the EIB board during surgical implantation. **(E)** The stationary probe for chronic recordings. During surgery the probe is covered in dental cement for protection. Supports may be added to the front or back of the implant if needed. Thirty-two-channel single unit probes and LFP probes use the same EIB and therefore will both look like example. See Supplementary Videos for additional information on probe fabrication.

Insulated metal wires are commonly used as the voltage-collecting interface in single unit recordings. Many different sizes (5–50 μm diameter) and types of wires (including tungsten, stainless steel, and nichrome) have been used to collect single units (54, 71, 72). Wire size influences the number of neurons recorded simultaneously, signal-to-noise ratio, and electrode impedance levels (73, 74). To optimize for longevity, we designed probes with thin nichrome wires and bundled them together in a brush-like design to facilitate implantation by minimizing damage of brain tissue (40–42, 44). Fixing the microwires in bundles provides some control of the spatial arrangement that can be lost with a standard brush electrode (43). After testing various diameter wires, we used 12.7 μm diameter wires (Sandvik, Stockholm, Sweden), the thinnest we could still implant into the brain. Typically, such thin wires are twisted into a stereo-trode or tetrode in order to make them stiffer, though the consequence of twisting is an overall greater diameter of the final probe. In our approach, each wire was cut (using tungsten-carbide micro dissecting scissors; Stoelting, Wood Dale, IL, USA) into 5–6 cm segments. Exact wire length depends on depth of target location. Wires with any sign of bending were discarded. We used several steps to increase the overall strength of these wires for the purpose of implantation. First, we inserted eight wires into a metal cannula, creating significant structural support for each wire. Cannula was fabricated using 30-gauge stainless steel tubing (Mcmaster-Carr, Elmhurst, IL, USA) cut into 8–9 mm sections, with the lumen cleaned with cleaning wires (Hamilton Company, Reno, NV, USA) and secured with electrical tape to a glass microscope slide for a clean, level surface (Supplementary Videos 1–3). Eight pieces of cut wire were pulled through the proximal end of one metal cannula with precision tweezers or forceps (Fine Science Tools, Foster City, CA, USA) with at least 10 mm extending beyond the distal end. Wires on the distal end were carefully separated into two small bundles, each containing four wires (Figure 1A). A tiny amount of superglue (Loctite, Dusseldorf, Germany) was applied using a 30-gauge hypodermic needle tip (Exelint International Co., Redondo Beach, CA, USA) to hold the bundles separate from each other without covering the electrode tips. Each bundle of four wires was separated again into two bundles of two wires and held in place with superglue (Figure 1A). Wire at the distal end was fixed to the metal cannula by superglue and was trimmed with serrated scissors (Fine Science Tools) to extend 6 mm beyond the cannula opening (Supplementary Videos 1–3). The exact length of wire extending beyond the cannula should be adjusted based on brain location depth.

Two of these assembled eight-wire cannulas were aligned together by matching the distal and proximal cannula ends and glued together to form a 16-wire, two cannula bundle (Figure 1A; Supplementary Videos 1–3). Glue was allowed 10 min to dry, after which two 16-wire bundles were aligned and glued to form a 32-wire bundle with electrode tips spreading across an area of ~1 × 1 mm (Figure 1A). This approach is scalable, and thus while 32-channel probes were utilized for most surgeries, we fabricated 64-channel probes in some animals, achieved by aligning and securing two 32-wire bundles. The positioning (ML/AP coordinates) of each 32-wire bundle and the length of their wires (DV coordinate) could be measured to target multiple areas of interest. Each cannula is held in place with alligator clips mounted to a “helping hands” stand (Amazon, Seattle, WA, USA) or stereotaxic arm and adjusted to the desired distance from each other where they are held in place with dental cement and superglue to allow for fast drying (Supplementary Videos 1–3). Our probes were designed to target either anterior cingulate cortex (ACC; AP: +3.2 mm, ML: ±1.0 mm, DV: 3.5 mm), ventral orbitofrontal cortex (VO; AP: +3.5 mm; ML: ±1.5 mm; DV: 5.0 mm), and/or lateral orbitofrontal cortex (LO; AP: +3.5 mm; ML: ±2.5 mm; DV: 5.0 mm) (Table 2). Although we targeted regions within one hemisphere, the design is flexible and can be adjusted to target locations in both hemispheres. The cannula are then mounted on a 32-channel (Neuralynx, Bozeman, MT, USA) or 64-channel (Open Ephys, Cambridge, MA, USA) omnetics-connected EIB board (Figure 1A) (76). The EIB board was secured and cannulas held in place with electrical tape onto a “helping hands” arm or stereotaxic arm with electrode tips (to be inserted into the brain) furthest from the EIB. Wires extending from cannulas were threaded through channel holes on the inferior side of the board one at a time with precision tweezers (Open Ephys). Wires were secured in channel holes with gold pins (Neuralynx/ Open Ephys) clamped in place with serrated jaw pliers (Open Ephys) (Supplementary Video 3). In our experience, if gold pins are secured correctly, they make a better connection than solder or silver paint. This process continued until all wires (32 or 64) were threaded through the EIB holes and channel mapping pattern was noted (Ex. Electrode 1 in Channel 0). Superglue and dental cement were carefully placed to hold cannula to the EIB board. These materials are preferred as their impedance should not interfere with the signal as can be seen with other materials. Care was given to leave the EIB omnetics attachment and ground hole (ground wire is secured during implantation) free of glue/ dental cement. Each probe was made 2–3 days before scheduled implantation. The probe was stored in a covered tray with a small piece of putty placed at the edge of EIB to hold the probe in place and avoid damaging electrode tips. On the day before surgery, wire tips were electroplated to an impedance of 1 MOhm at 1 kHz using the nanoZ impedance tester (Neuralynx) and gold-plating solution (Neuralynx) (see nanoZ manual for details). The electrode tips were dipped in a small beaker of 70% ethanol for 1 minute and gently dried with Kimwipes (Kimberly-Clark, Fisher Scientific). The assembled 32-channel probe has a size of 20 × 19 × 25 mm and a final weight of 1.5–2 g. Fabrication of a 32-wire bundle (two, 32-wire bundles in a 64-channel electrode) can be finished within 2–3 h by a trained person, while attaching and pinning of the wire bundle to the EIB board takes another 2–3 h.

**Table 2:** Anatomical targets and surgical coordinates of single unit and local field potential probes.

**AP**	**ML**	**DV**	**Target area**
**SINGLE UNIT TARGET LOCATIONS**			
3.2	1.0	3.5	Anterior cingulate cortex
3.5	1.5	5.0	Ventral orbitofrontal cortex
3.5	2.5	5.0	Lateral orbitofrontal cortex
**LOCAL FIELD POTENTIAL TARGET LOCATIONS**			
**Cannula 1**			
3.75	0.8	0.8	M2
3.75	0.8	3.2	A32D
3.75	0.8	4.8	A32V
3.75	0.8	5.8	Ventral orbitofrontal cortex
**Cannula 2**			
3.75	3.2	1.0	Anterolateral motor cortex
3.75	3.2	3.6	Lateral frontal cortex
3.75	3.2	4.8	Anterior insula
3.75	3.2	5.8	Lateral orbitofrontal cortex
**Cannula 3**			
2.0	0.6	2.0	A24b
2.0	0.6	3.0	A24a
2.0	0.6	3.5	A33
2.0	0.6	6.6	Nucleus accumbens shell
**Cannula 4**			
2.0	1.8	1.5	M2
2.0	1.8	4.5	Dorsomedial striatum
2.0	1.8	5.7	Ventromedial striatum
2.0	1.8	6.9	Nucleus accumbens core
**Cannula 5**			
−2.5	0.7	1.3	A30
−2.5	0.7	2.3	A29
−2.5	0.7	4.7	Mediodorsal thalamus
−2.5	0.7	5.7	Centro-median thalamus
**Cannula 6**			
−2.5	4.9	5.1	Dorsolateral striatum
−2.5	4.9	6.1	Dorsolateral striatum
−2.5	4.9	7.1	Central amygdala
−2.5	4.9	8.1	Basolateral amygdala
**Cannula 7**			
−3.5	2.5	1.4	Posterior parietal cortex
−3.5	2.5	2.5	CA1
−3.5	2.5	3.5	CA3
−3.5	2.5	8.0	Subthalamic nucleus
**Cannula 8**			
−6.0	3.5	1.0	V1
−6.0	3.5	1.7	V1
−6.0	3.5	2.8	Dorsal subiculum
−6.0	3.5	3.7	Dentate gyrus

*The table lists AP, ML, and DV coordinates in mm for each location. All coordinates are relative to bregma. Nomenclature is consistent with (75)*.

### Local Field Potential Probe Design

For LFP probes we selected 50 μm tungsten wire precut to 10 cm length wires (California Fine Wire, Grover Beach, CA). Precut wires greatly facilitated standardization of this process and were much straighter than spooled wire. We chose 50 μm wire to capture field potentials due to the lower impedance (which is helpful for recording lower frequencies). Although the thickness increases the chance of damage and inflammation upon implantation, only one electrode is placed at each site, minimizing local damage compared to larger-footprint polytrodes/ tetrodes. Four of these wires were bundled and inserted into a 30-gauge stainless steel cannula (Mcmaster-Carr) cut 8–9 mm in length (Figure 1B). Each wire was arranged according to the desired DV measurement of the target brain region using precision tweezers or forceps (Fine Science Tools) to carefully manipulate the wires and avoid bending (bent wires were discarded) (Supplementary Videos 4–5). Each cannula was designed to target four different depths at the same AP and ML location. Each cannula was thus optimized for a particular AP/ML site, with electrodes adjusted to different lengths by measuring the amount of wire extending from the distal end of the cannula (Figure 1B; Table 2). Wire lengths extending from the cannula range from 2 to 9 mm and were measured to account for the space between skull and cortical surface, to prevent cannulas from extending beyond the skull surface. Having a standard approach for inserting and marking wires is important to map each wire onto an EIB channel. We suggest either cutting the proximal ends of the wire to represent shortest-longest distal wire lengths, or color coding each wire small pieces of colored electrical tape. After electrodes were adjusted to length, a small dot of superglue was applied to the inside of tubing via capillary action using a 30-gauge hypodermic needle (Exelint International, Co.) with caution to secure the wires in place without covering the electrode tips with glue (Supplementary Videos 4–5). Each cannula was 0.5 mm in diameter, with the four protruding wire tips covering an area ~ 0.12 mm wide. Electrode bundles can be prepared in bulk and stored securely in a covered drawer/tray for months at a time before use. Eight sets of such electrodes were prepared, labeled (1–8) and sterilized in an autoclave (Figure 1B). Electrodes for field potential recordings were not electroplated. In our standard approach, all 32 wires were implanted in one hemisphere, but the versatility of our design allows researchers to target different brain regions of interest within or across hemispheres. The rest of the assembly (inserting wires and securing into EIB) occurred during implantation surgery and is described below. A trained individual can build eight probes in 1–2 h. A finished probe with all eight cannula pinned to an EIB board weighs 0.7 g. When covered in protective layers of dental cement attached to ground and anchor screws, the probe weighs ~7.25 g and is ~22 × 27 × 20 mm in size.

### Ground Wires

Ground wires for single unit and LFP probes are soldered to a ground screw and autoclaved before being connected to the EIB during implantation surgery. Ground wire should be thick and flexible, ensuring a good connection. Therefore, we elected to use annealed stainless-steel wire with a diameter of 75 μm cut ~10 cm long (A-M Systems, Loop Sequim, WA, USA). The ground wire was soldered to a 5.20 mm-long, 1.15 mm-wide self-tapping stainless steel bone screw (Fine Science Tools). Insulation on one end of the wire was stripped by fine forceps (Fine Science Tools) and coiled around the threadless base of the screw. 1–2 drops of phosphoric acid was applied to the contact surface with a 30-gauge needle tip (Exelint International, Co.) to facilitate the soldering process. Wire solder 0.03″ diameter (Kester, Itasca, IL, USA) was then applied to the same area with precaution to only minimally cover the threaded surface.

## Methods

### Ethics Statement

This research was conducted in strict accordance with the Guide for the Care and Use of Laboratory Animals of the National Institutes of Health. The protocol was approved by the San Diego VA Medical Center Institutional Animal Care and Use Committee (IACUC, Protocol Number A17-014).

### Subjects

Our stationary probes designed for chronic *in vivo* electrophysiology were implanted in 39 male Long Evans rats obtained from Charles River Laboratories (recording single unit *N* = 9; local field potential *N* = 30). Rats were around 1 month old weighing 150 g when received and were given 2 weeks to acclimate before initiating behavioral training. Two rats were housed per standard plastic tub (10 × 10.75 × 19.5 in, Allentown, NJ, USA) prior to surgery, and single-housed following surgery. Rats were kept on a 12 h light/dark cycle (lights on at 6 a.m.) and tested during the light cycle. Food was provided *ad libitum* and water was restricted to 20 mL in a scheduled 1-h consummatory period on days with behavioral testing so that water could be used as a positive reinforcer during operant tasks. Water was unrestricted on non-training days. Depending on the behavioral study, rats were implanted prior to any behavioral training or implanted after performance met a criterion on basic training. Rats were 3–5 months old weighing 300–600 g at the start of recording. Subjects with chronic implants were monitored daily for signs of infections, injuries, and bleeding. Rats were 7–20 months old at the conclusion of study.

### Surgical Procedures

Stereotaxic surgery with sterile methods was used to implant both types of probes. All surgical tools were autoclaved prior to surgery. Rats were deeply anesthetized in an induction chamber with 5% isoflurane/ 96% room air using a low-flow anesthetic machine (Kent Scientific, Torrington, CT, USA). Rats were transferred to the stereotaxic frame and 1.9–2.5% isoflurane was delivered through a nose cone with the rat in a fixed position for the remainder of surgery. A body temperature-controlled heating mat (VWR, Radnor, PA, USA) was used to maintain temperature at 37 °C. Animals received a single dose of Atropine (0.05 mg/kg) to diminish respiratory secretions during surgery, a single dose of Dexamethasone (0.5 mg/kg) to decrease inflammation, and 1 mL of 0.9% sodium chloride solution. The area of incision was cleaned with 70% ethanol and iodine solution. Lidocaine was injected (0.2 cc max) to provide local anesthetic at the injection site. An incision was made to reveal skull bone and skin was held at the periphery by four skin clamps. Cranial holes for anchor and ground screws were drilled at the periphery, anterior, and posterior sides of exposed skull using 0.7 mm micro drills (Stoelting). The autoclaved ground screw and wire was tapped into the bone posterior to the lambda point on the skull, and a minimum of three anchor screws were tapped into the bone at the periphery of the skull. Screws were secured with CandB Metabond (Parkell, Inc., Edgewood, NY, USA) on dried skull bone.

#### Implantation of Single Unit Probes

A 2 mm diameter cranial window was drilled with a 0.7 mm micro drill (Stoelting) centered at the target AP/ML location for ACC, vOFC, or lOFC (Figure 1C, Table 2). Creating a cranial window large enough for our wires organized in a brush-like formation increases flexibility during implantation, as the optimal implantation site could be hindered by blood vessels. The cranial window is drilled down into a thin layer using sterile saline as needed to cool the skull until a thin bone flap remains which can be gently lifted away from the skull with forceps. The electrode tips of the single unit probe were soaked in a small beaker of 70% ethanol solution for 5 min prior to surgery. After removal of dura, the single unit probe was clamped in an alligator clip attached to a stereotaxic arm to position the electrode tips above the craniotomy and slowly inserted into the brain at a speed of ~5 μm/s (Figure 1C). Wires were lowered in areas with as few visible blood vessels as possible to avoid hemorrhage during implantation, as we found empirically that large hemorrhage during the implantation is often linked to rapid deterioration of signal quality. Drops of saline were used to fill the cranial window, and a thin layer of superglue was applied to the skull surface to seal the craniotomy. Dental cement was then applied over the superglue layer to further isolate the external environment from the cranial window. The implant was secured to anchor screws and attached to the dry skull by Metabond (Parkell Inc.) and the remaining exposed wires covered in dental cement.

#### Implantation of LFP Probe

Initial preparation of the animal and location of ground screw was identical to the single unit probe surgical procedures. Instead of a cranial window, eight small holes were drilled using a 0.5 mm micro drill (Stoelting) at predetermined stereotactic locations where LFP electrodes would be inserted (Table 2). A hypodermic needle (30 gauge) (Exelint International Co.) was bent using needle holders and used to carefully tear the dura to reduce wire damage that may occur with insertion. Holes were filled with a small drop of saline. Electrodes are built in bundles each containing four wires with different DV lengths. Eight of such bundles were implanted at different AP and ML locations, making a total of 32 LFP targets throughout the brain. See Table 2 for exact coordinates and list of brain targets used. Electrode sites were chosen to target as many potential brain sites as possible across the D/V axis while minimizing the number of cannulas (and therefore holes drilled). We have found two successful ways to implant the 32-channel LFP probes, each described in further detail. (1) Each cannula is attached one at a time to a stereotaxic arm, lowered into the brain, and then attached to the EIB board. Under stereotactic control, electrodes were slowly lowered at ~5 μm/s to desired depth centered over the drilled hole and measured such that the cannula did not enter the brain. We suggest the surgeon use a microscope to watch wires as they enter the brain to ensure they do not bend if there is resistance and to guarantee all wires are centered in the drilled hole. Once lowered to the target location, the bundle is secured to the skull with superglue (Loctite) and covered with Metabond (Parkell), cautious not to cover other open electrode holes. This procedure is repeated for all eight bundles. Once all eight cannulas are placed and secured, visible wires extending from the top of the cannula are threaded through the holes of a 36-channel EIB (Neuralynx) and held in place using gold pins (Neuralynx) clamped down with serrated jaw pliers (Open Ephys) (Figure 1D). To do this, the EIB is attached to a stereotactic arm and lowered to a position that is centered at least 1/2 inch above the cannulas. The experimenter needs room to manipulate and thread the wires through the inferior end of the EIB and secure with gold pins (as described previously). This procedure should be done systematically as the experimenter will need to note exactly which wire (shortest-longest) was threaded through each EIB channel. Extra wire that is not broken off while clamping is cut flush to the pins. The stainless-steel ground wire (same as single unit probe) is also pinned in place. The entire apparatus is covered in dental cement to protect the wires and EIB board. (2) An alternative method for implanting the LFP probe includes first connecting the wires to the EIB channels outside of the surgical field and then lowering all eight cannulas into the brain simultaneously. In this method, the EIB board is held in place by an alligator clip attached to a “helping hands” adjustable stand (Amazon). One at a time, each cannula is also held in place with an adjustable arm and its four wires are each threaded through a channel hole on the EIB board and pinned to the EIB (described previously). This is done systematically to note which wire (shortest-longest) is inserted into which EIB channel. A small drop of dental cement is applied with a hypodermic needle to the bottom of the channel hole, to further secure the pin and wire without covering other open channel holes. This procedure is repeated until all eight cannulas are secured to the EIB. The placement of each cannula should be measured relative to the other cannulas according to the desired AP/ML measurements before being pinned in place and secured with cement. If needed, a small strip of electrical tape can be used to hold cannulas along the same AP axis together at the correct distance apart. Once all cannulas are in place more dental cement is added to the bottom and top of the EIB to cover the pinned connections, with care to leave the ground wire hole open. The cannulas are soaked in a small beaker of 70% ethanol for 5 min before the EIB board is attached to a stereotaxic arm. Centering the EIB and cannulas over the drilled holes, the implant is lowered at ~5μm/s. You can measure the DV of the longest wire at the skull surface as reference for how far to lower the implant or rely on the measurements from the cannula bottom (cannula should sit flush with skull surface). We recommend using a microscope to visualize wire implantation. Wires may need to be guided into place using a small needle or forceps. EIB must stay level for cannulas to travel synchronously. Once in place, the skull surface is dried with a fine cotton swab or Kimwipe (Kimberly-Clark, Fisher Scientific), a small dot of superglue is applied around each hole, and dental cement is used to flood the skull surface around all cannulas. The ground wire is inserted and pinned into its corresponding channel on the EIB and the implant is covered in dental cement (Figure 1D). This second approach saves a substantial amount of time during surgery but restricts the ability to adjust each individual cannula. The first approach does require manipulations (pinning wires to EIB board) after wires are implanted, placing stress on the implant which may cause micro-movement of wires in the brain. A combination of both approaches (i.e., fixing some cannulas to EIB before surgery) may also be suitable depending on experimental design and brain targets.

When the dental cement is fixed for both single unit and LFP probes, the skin is sutured closed using non-absorbable sutures (Stoelting). Animals are given a one-time dose (1 mg/kg) of long-acting buprenorphine (72 h) post-surgery. The rat is returned to the home cage and monitored until awake and ambulatory. A heating pad is placed under the home cage to prevent hypothermia during recovery. Sulfamethoxazole/Trimethoprim (60 mg/kg) was given in drinking water for 7 days post-surgery to prevent infection. Figure 1E shows a finished 32-channel implant for chronic recording.

### Electrophysiology Recording

**Figure 2:**
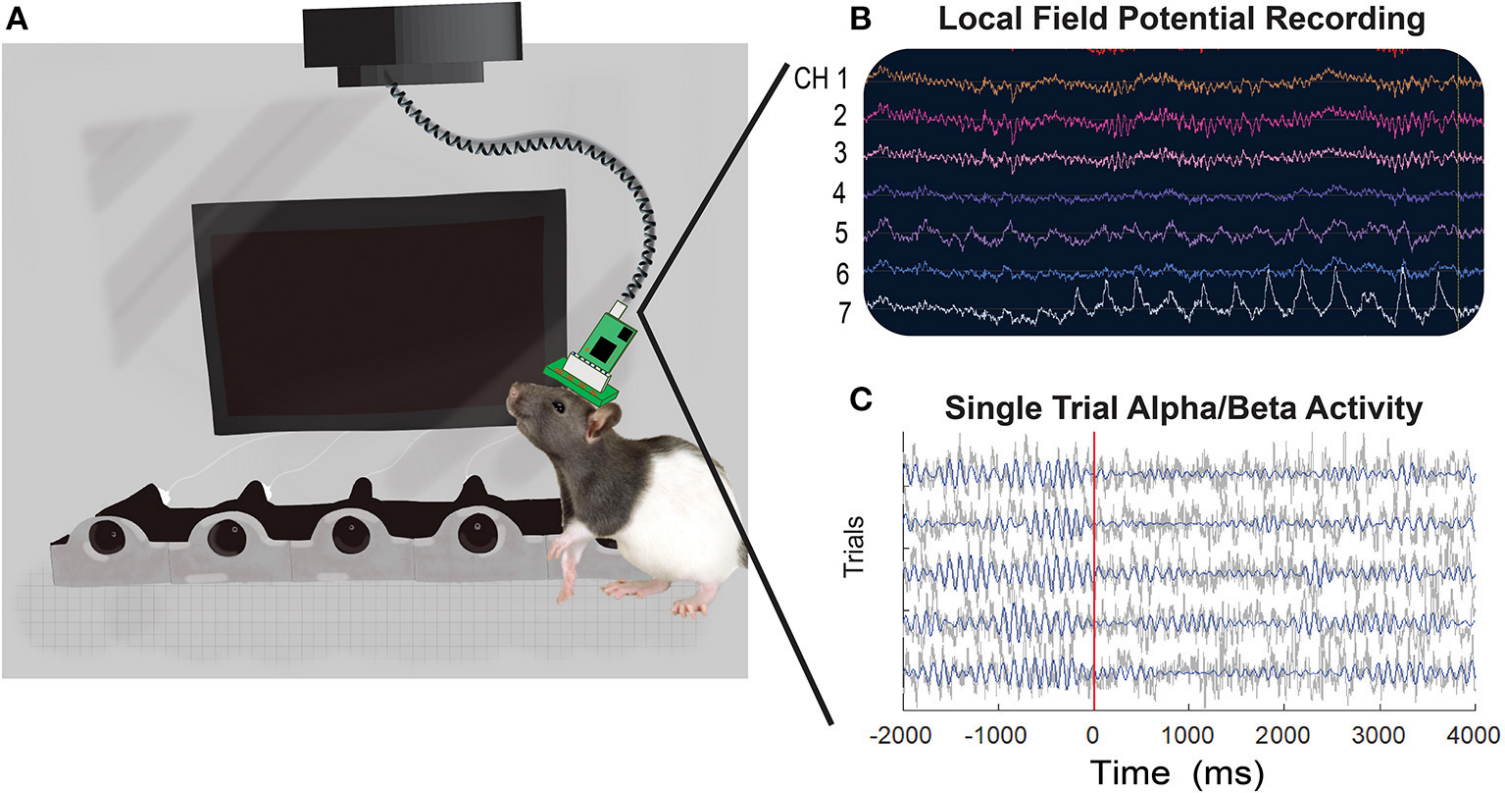
An illustrative representation of an implanted rat in the operant chamber with examples of local field potential activity recorded during a visual discrimination task. **(A)** Rats are recorded in an operant chamber equipped with five noseports with LED lights, a visual screen, and five motors to deliver water through noseports. The rats' EIB board (32 or 64-channel) is connected to a RHD headstage and interface cable (Intan Technologies) mounted to a commutator (TDT). Single unit data is collected through TDT devices and recorded with Synapse software, whereas LFP data is collected through Intan devices and processed with Open Ephys software. **(B)** An example of raw LFP traces across seven of the 32 channels from a single recording session is shown. **(C)** Example LFP traces from five trials recorded from electrode A29C. Gray lines show the raw time-series and blue lines show the 8–20 Hz (alpha/beta) filtered activity.

Rats were recorded in a 6.2 × 4.7 × 6.23 inch operant chamber with a ceiling opening to allow for electrophysiology cables to move freely. The chamber and software control we developed and used for this and other studies as described previously (77, 78). A Faraday cage was built around each chamber, and the chamber was powered using 24 V batteries (TalentCell PB24A1 72 W battery, Amazon) to reduce electrical (60 Hz) noise. Each RHD 32-channel recording headstage is connected to a RHD SPI Interface cable (Intan Technologies, Los Angeles, CA, USA), shielded to protect cable from damage during recording sessions. The interface cables both attach to a grounded motorized commutator (TDT, Alachua, FL, USA) (Figure 2A).

The signal from our single unit probes is amplified by the PZ5 Neurodigitizer and RZ2 Bioamp Processor (TDT). Recorded signals are processed using Synapse software (TDT) at a sampling rate of 25 KHz. The high-pass filter is set at 300 Hz and the low-pass filter at 3,000 Hz. Behavioral markers from operant tasks are sent to the Synapse software via lab-streaming layer (LSL) software (25) to integrate and store physiological and behavioral data streams. Raw data is stored as a tev file.

The signal from our LFP probes was recorded using a 32-channel RHD headstage (Part C3324, Intantech) connected to a grounded Intan RHD2000 Evaluation Board (Intantech, Part C3100) with an SPI interface cable. The Rhythm FPGA, part of Open Ephys plugin-GUI, reads data from the Evaluation board (76). The physiology data is processed through Open Ephys using a sample rate of 1.0 Ks/s at a bandwidth of 0.1–999.4 Hz and DSP of 0.3 (Figures 2B,C) (76). Behavioral markers are also captured via LSL software using a customized plug-in written for the plug-in GUI (https://github.com/aojeda/plugin-GUI) and stored as an xdf file. See Figures 2B,C for an example of raw LFP traces. The TDT and Intan setups are both capable of recording single unit and local field potential data. We had both systems available and therefore dedicated TDT for single units and Intan for LFP, but it is not necessary to acquire both systems. Recording sessions last 60 min. On average, rats were recorded three times a week.

### Behavioral Training

The full details of our operant tasks and the accompanying electrophysiological results have been described previously (79). Here we briefly describe one of our behavioral tasks to demonstrate how our probes can be utilized to study cognitive behaviors in rodents and to show the quality of physiological signals captured. All behavioral training and testing occurs in custom built behavioral chambers with screen to display visual stimuli and five noseports each with LEDs and water spouts controlled by motors (77) (Figure 2A). A test of motor inhibition, the visual discrimination go/wait task requires rats to respond appropriately (“go” or “wait”) based on a visual cue. During a “go” trial (vertical rocket with stripes) the rat has 2 s to respond resulting in water reward. An error (if the rat failed to respond within 2 s) results in a time-out period and no water delivery. On a “wait” trial (horizontal white rocket) the rat must withhold its response for 2 s. Correctly withholding followed by a response results in water reward whereas failure to withhold results in an error. Trials were distributed as 75% “wait” and 25% “go” to capture the action delay behavior which is harder to perform correctly. Physiological activity is time-locked to trial onset or response. We typically waited 1–2 weeks after surgery before resuming behavioral testing and electrophysiology recording.

### Data Analyses

#### Single Unit Analyses

Recorded data were cleaned and referenced off-line using Wave_Clus v.2.5., a Matlab-based spike-sorting program (80, 81). With brush electrodes the standard is to sort activity on each channel (44, 82, 83). Although our probes are designed as single channel recording sites, the fan-like arrangement of wires may cause spikes to be detected on multiple recording sites within each fixed cannula bundle. Obaid et al. (83) found that even with wires spaced 100 μm apart, each single unit detected was recorded on ~1.4 different sites. Therefore, we use “polytrode” sorting to compare spikes captured by 16 wires in a fixed bundle to provide a conservative metric and avoid a bias cell count driven from units that may have been recorded on multiple channels. Our probes are not arranged like a tetrode (four recording sites 25–50 μm apart) or polytrode (8–64 channels 50–70 μm apart) with fixed distances to capture multiple views of the same neuron, but since the goal of clustering is to find similarity between spikes we can sort in multiple channels, acknowledging that we are not sorting any better than a single microwire (54, 73, 80, 81, 84–86). Signals were processed as 16 electrode groups during referencing (two polytrode groups for 32-channel probes; four polytrode groups for 64-channel probes) selected because of their fixed arrangement into different cannula during fabrication. The quality of spike detection depends on isolating spike amplitudes compared to background noise (73), thus median referencing against the 16-trode group was applied to each channel. There are three basic stages to single unit analysis: spike detection, feature extraction, and clustering (73, 81, 84). First, a threshold of spike detection was set at 5 times standard deviation of voltage potential in each channel. Broken channels, with large impedances beyond 10 MOhm, were excluded from referencing or clustering. Second, Wave_Clus uses a wavelet transform to select the ideal coefficients for each of the identified spikes that give optimal separation between different clusters (73, 84). Finally, Wave_Clus uses a non-parametric clustering algorithm, super-pragmatic clustering, to group spikes into clusters (**Figures 5A–D**). Clustering of polytrodes is done by concatenating the spike shape collected in the 16 channels (87). The distinctive clusters in each individual channel were automatically compared by Wave_clus across other clusters in the polytrode group and merged with those having similar spike features. Spikes in each polytrode group (max of four polytrodes), as identified by Wave_Clus, were examined manually for characteristics of single units. Spikes were considered a single unit when the average spiking rate was more than 0.5 Hz across the recording session, had fewer than 1.5% inter-spike interval (ISI) violations (<3 ms), when waveforms resembled action potentials as opposed to sinusoidal noise artifacts and when the cluster was distinct from other clusters in the principal component space (**Figures 5A–D**) (73, 86, 87). Spikes meeting this criterion are counted as single units and time-locked with behavioral events. Using these criteria, we evaluated our probes on yield, efficiency, cluster count over time, and signal-to-noise ratio (SNR). These metrics are reported in Table 3 and compared against the performance of other commercial and custom electrophysiology arrays. Yield describes the number of neurons/ areas. We calculated yield separately for each rat and recording session in order to more accurately compare to other studies where yield would be dependent on total number of animals and recording sessions. Similarly, efficiency is the neurons/recording site (32 or 64) which was also calculated separately for each animal and session. Numbers are reported as averages and standard error of the mean (SEM). SNR was calculated for each unit as the peak-to-peak amplitude of the mean waveform divided by the root mean square (RMS) noise voltage (62, 70).

**Table 3:** Comparison of electrophysiology devices measuring extracellular single unit activity in rodents.

**Probe**	**Our probe**	**Brush microwire array**	**Fixed microwire array**	**Neuralynx microdrives**	**Open Ephys shuttle drive**	**Microprobes floating array**	**Utah intracranial electrode array**	**NeuroNexus michigan**	**Neuropixels**
		**Custom**	**TDT^**a**^; Custom^**b, c, d**^**	**Versa Drive 8^**a, b**^; Harlan 8^**c**^**					
Target Area (s)	Orbitofrontal cortex, anterior cingulate cortex	Trigeminal ganglion, thalamus	Somatosensory cortex^a,b,d^, auditiory cortex^c^, brainstem^b^, thalamus^b^	Cortical^a^, nucleus accumbens^b^, hippocampus^c^	Somatosensory cortex	Somatosensory cortex	Motor cortex	Cortex	Multi-site forebrain and midbrain
Number of Sites	32–64	3–7	14–48	16^b^-32^a,c^	16–64	16	16–128	16	384 per probe
Cost	150 USD	–	150^d^-295 USD^a^	280^b^-1,900^c^ USD	500 USD	1,100 USD	3,500 USD	–	1,000^a^ USD
Assembly time	4–6 h	–	–	1.5^b^-5 h^a^	<1 day	–	–	–	3 h^a^
Longevity	168 days (56 average)	80 days^b^	42^c^-273 days^a^	24^a^-40 days^c^	290 days (90 average)	21^b^-182 days^a^	84 days^a^	42^c^-382 days^b^	42^a^-147 days^b^
Yield (neurons per area)	5.82 ± 0.03, 7.32 ± 0.06	18 ± 1.3^b^	–	4.7–5.7^a^, 5.4^c^, 20^a^	10–20	–	5.92^a^	8.96 ± 0.6^c^	22.2^c^, 71.2^a^, 79^b^
Efficiency (neurons per recording site)	0.15 ± 0.01, 0.23 ± 0.01	0.14 ± 0.06^b^	2.3 ± 0.4^b^	1.05 ± 0.7^a^	1.43	0.625^b^	0.28^a^, 0.46^c^	1.0 ± 0.03^c^	0.19^a^, 1.5^c^
Signal-to- noise	11.74 ± 0.21	7.5^b^	3.7^b^, 6.0^c^, 11.0^d^	8.69 ± 0.24^a^	–	3–6^a^	10.35^a^, 6.1^b^	7.07 ± 0.47^a^, 4.3 ± 1.0^c^	5.05^a^, 8.78^b^
Sources		Tseng et al., 2011^a^; (59)^b^	(60)^a^; (61)^b^; (62)^c^; (63)^d^	(64)^a^; (55)^b^; (51)^c^	(65)	(66)^a^; (67)^b^	(50)^a^; (49)^b^; (45)^c^	(68)^a^; (69)^b^; (70)^c^	(46–48)^a,b,c^

*Numbers are presented as averages and SEM unless otherwise stated*.

*Superscripts denote the source of information*.

#### LFP Analyses

To measure neural activity in brain regions linked to time-locked task events we performed standard pre-processing and time-frequency (TF) analyses using MATLAB and functions of EEEGLAB. Pre-processing steps include identifying and time-locking neural activity with behavioral markers of interest, removing trials with large artifacts, referencing each channel to the median across all channels and then performing TF decomposition of neural activity using a complex wavelet function. First, we extracted time-points for events of interest (usually trial start and response) dependent on the behavioral task. To assemble the time-series data into a matrix with electrodes, times, and trials we used data epoching. Next, a standard deviation was calculated for each trial. Standard deviation was calculated for each electrode across time in a particular trial and then averaged across electrodes. If the standard deviation of a single trial was >4 times the mean standard deviation of all trials it was considered “noisy” and discarded. Activity was then median referenced for each time-point by calculating the “median” activity across all electrodes and subtracted that “median” from each electrode. TF decompositions are calculated using a complex wavelet function implemented within EEGLAB (newtimef function, using Morlet wavelets, with cycles parameter set to: [2, 0.7], frequency window of between 2 to 70 Hz and otherwise default settings used). Analytic amplitude of the signal was calculated using the abs function. For each channel/frequency the mean activity within a baseline window (prior to the start of a trial) was subtracted to measure evoked activity (change from baseline). We next calculated the average activity for specific trial types (“go” correct, “wait” correct or “wait” incorrect in the go/wait task) at each time-point and frequency for each electrode, thus creating a 3D matrix (time, frequency and electrode) for each behavioral session. Before averaging across sessions/ animals, data was “z-scored” by calculating the mean and standard deviation for each electrode at each frequency across time. Z-scoring was helpful in normalizing activity measured from different animals/sessions and was important particularly in minimizing the effect of certain outlier animals. Pre-processing resulted in a 3D time-frequency-electrode matrix for each session that could be used in further statistical analyses. Figure 2C provides an example of individual trial activity for one electrode (A29C) time-locked to task events and filtered for alpha/beta activity.

### Histological Analyses

**Figure 3:**
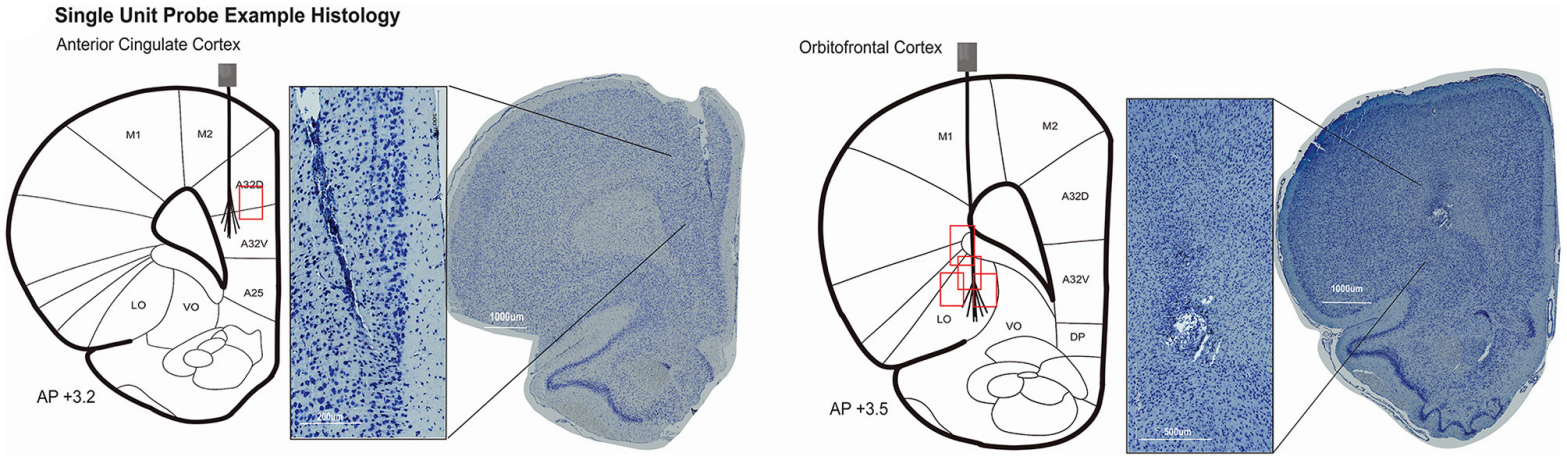
Histology was completed in 6/9 rats with single unit probes. Evidence of the electrode location could not be identified in one rat. Coronal rat brain sections showing brush-electrodes in ACC (*N* = 1) and OFC (*N* = 4). Track locations visualized with Zen lite software (Zeiss) from slide scanner images at 40× magnification. Schematics of electrode targets and recording locations are plotted on a modified rat brain atlas [adapted from (75)]. Red boxes represent the estimated area of recording compared to the target location of 32–64 channel brush microelectrode. Evidence of the ACC electrode track was seen from 3.2 to 2.5 mm anterior to bregma (shown at + 3.2 mm AP). OFC electrodes ranged from 4.0 to 2.5 mm anterior to bregma (shown at + 3.5 mm AP).

**Figure 4:**
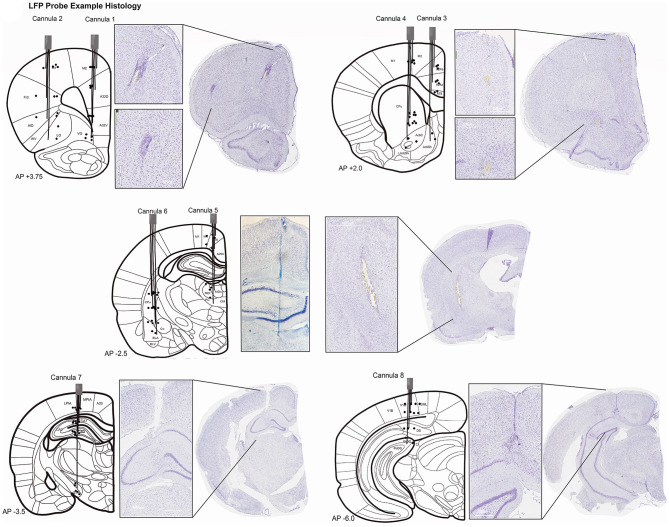
Histopathology for LFP recording sites. Histology was completed for 10/30 rats with LFP probes. Each rat had electrodes targeting all 32 coordinates. Sections were visualized from a slide scanner at 40× magnification with Aperio Imagine Scope (Leica Biosystems). Schematics of electrode targets and actual locations are plotted on a modified rat brain atlas [adapted from (75)] for all eight cannulae. Black dots represent the electrode depth estimated for the four wires in one cannula. Not all electrodes could be visualized for every rat. Evidence of Cannula 1 and 2 tracks was seen starting at +4.5 mm anterior to bregma through +3.0 mm (shown at +3.75 mm). Histopathology for Cannula 3 and 4. Evidence of Cannula 3 was observed in coronal brain sections starting at +2.5 mm anterior to bregma and was seen through +0.5 mm. Cannula 4 also started at +2.5mm but was observed through −1.0 mm bregma. Both are shown at AP +2.0 mm. Histopathology for Cannula 5 and 6. Cannula 5 and 6 electrode tracks were first seen −1.5mm from bregma and were seen through −3.5 mm in one rat. A whole brain image was not able to be captured for Cannula 5, but a close-up example of the track is still shown. Both tracks are plotted on a coronal section at AP −2.5 mm. Histopathology for Cannula 7. Evidence of Cannula 7 began as early as −2.5 mm and was seen −4.5 mm posterior to bregma. Cannula 7 is shown at AP −3.5 mm. Histopathology for Cannula 8. Electrode locations of Cannula 8 is plotted at AP −6.0 mm but the track was seen as early as −4.5 mm from bregma through −6.0 mm. Terminology is consistent with (75). A24, A25, A29, A30, A32D, A32V, A33, cingulate cortex; AcbC, accumbens nucleus core; AcbSh, accumbens nucleus shell; AID, AIV, agranular insular cortex; BLA, BLV, basolateral amygdaloid nucleus; Ce, central amygdaloid; CA1, CA2, CA3, hippocampus; CM, central medial thalamic nucleus; CPu, caudate putamen; DG, dentate gyrus; DP, dorsal peduncular cortex; DS, dorsal subiculum; Fr3, frontal cortex; LO, lateral orbital cortex; M1, primary motor cortex; M2, secondary motor cortex; MD, mediodorsal thalamic nucleus; PC, paracentral thalamic nucleus; Pta, parietal association cortex; STh, subthalamic nucleus; V1, primary visual cortex; V2, secondary visual cortex; VO, ventral orbital cortex.

At completion of the study, single unit electrode tips were marked by passing 12 μA current for 10 s through each channel using the Nano-Z (Neuralynx) (not done on field potential probes). Rats were sacrificed under deep anesthesia (100 mg/kg ketamine, 10 mg/kg xylazine IP) by transcardiac perfusion of physiological saline followed by 4% formalin. Brains were extracted and immersed in 4% formalin 24 h and then stored in 30% sucrose 4% formalin until ready to be sectioned. Tissue was blocked in the flat skull position using a brain matrix (RWD Life Science Inc., San Diego, CA, USA). Six brains were sectioned frozen in the coronal plane at 50 μm. Eleven brains were paraffin embedded and sectioned 20 μm thick (processed by Tissue Technology Shared Resource; CCSG Grant P30CA23100). Brain slices were stained for Nissl bodies using thionin. Sections were processed with a slide scanner at 40× magnification (Zeiss, Oberkochenn, Germany; Leica Biosystems, Buffalo Grove, IL, USA) to identify the course of electrode tracks in target brain areas for both single unit and LFP probes. Positions of electrodes were inferred by matching landmarks in sections to the rat atlas when electrode tips could not be identified (Figures 3, 4).

## Results

**Figure 5:**
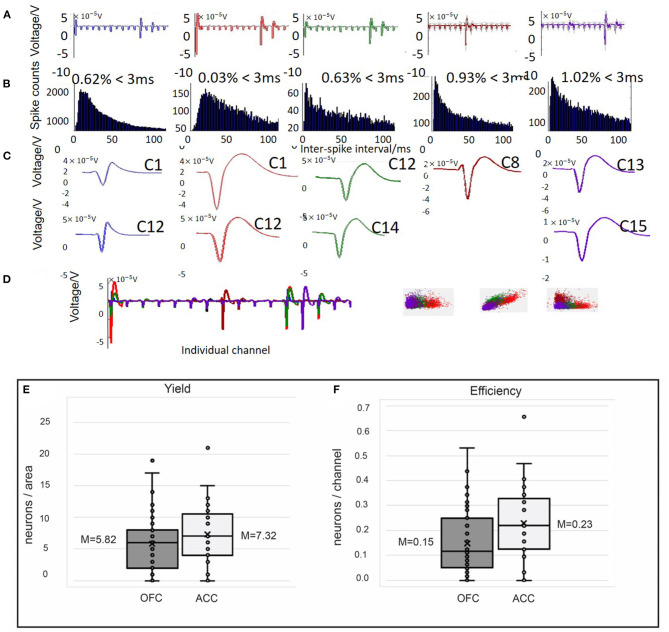
Example spike sorting data from 1 day of recording in OFC **(A–D)** and yield **(E)** and efficiency **(F)** obtained from single unit recordings in both OFC and ACC. Spikes detected from single channels were sorted offline using Wave_Clus software. **(A–C)** Five clusters (different colors) identified by Wave_Clus from one “polytrode” (16 channel bundle). **(A)** The average waveform is shown on each channel (1–16). **(B)** Spike counts and interspike interval histograms for each cluster identified. Only clusters with ISI violations under 1.5% (<1.5% of spikes occur within 3 ms) were considered single units. **(C)** The average waveform on different channels (i.e., C1 = channel 1) across the five identified clusters. **(D)** Average waveforms of all five clusters (different colors) found on channels 1–16 and the identified clusters separated in k-means space. Using these criteria to classify single units, we evaluated the yield **(E)** and efficiency **(F)** of our single unit probes, shown as box and whisker plots with mean (x) and median marked. **(E)** Here, yield is calculated as neurons/ area for each animal and recording session. In OFC our average yield was 5.82 compared to 7.32 neurons/area in ACC. **(F)** Efficiency, the number of neurons collected/ channel for each animal and recording session, was on average 0.15 in OFC and 0.23 in ACC.

**Figure 6:**
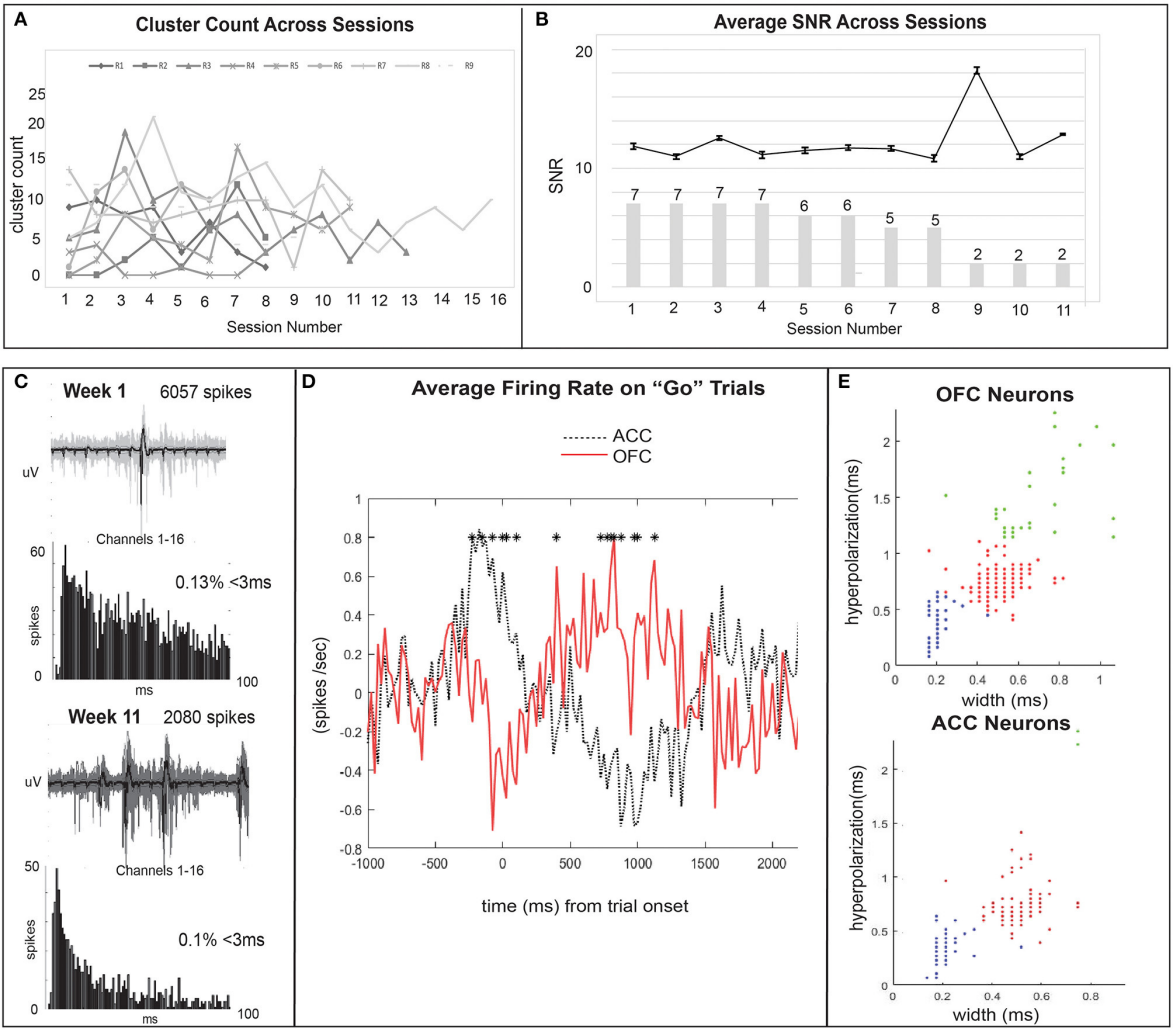
Quality units are further analyzed and time-locked with behavioral task events. **(A)** The cluster count across sessions is plotted for each individual rat (*N* = 9) to show the ability of our probes to capture quality signal over a chronic time span. Cluster count (number of units per session) ranged from a maximum to 21 units to a minimum of 0 units recorded per session and generally remained stable across time. **(B)** The mean SNR of all OFC units (*N* = 7) is plotted across time (session number). Error bars show SEM values. Bar plot represents the number of rats with active electrodes at each session number. There is a reduction from 7 to 2 rats with working implants over 11 recording sessions (~12 weeks). **(C)** The single units collected over 12 weeks remained relatively stable across time as seen with cluster count across sessions and SNR. Here, an example waveform, number of spikes, and ISI histogram, are compared for OFC units from the same polytrode collected in week 1 and week 11 to also illustrate this point. We did not perform analyses to track units across time and therefore cannot determine if this is the same unit held across 10 weeks. **(D)** The average firing rate (spikes/sec) of all ACC (black dotted line) and OFC (red solid line) neurons during correct “go” trials on the Go/Wait task. Activity is baseline normalized by subtracting the average firing rate during a pre-response baseline (first 500 ms) from firing rate in subsequent time bins and time-locked to trial onset. Significant differences in activity (*p* < 0.05) from a paired samples *t*-test are shown with asterisks. **(E)** K-means clustering is used to group neurons based on their waveform characteristics as putatively excitatory or inhibitory. ACC and OFC neurons are sorted by their hyperpolarization duration (ms) and width (ms), measured as peak-to-trough duration. Blue dots are putatively fast-spiking inhibitory neurons (brief hyperpolarization and small width), red are excitatory (longer hyperpolarization and larger width), and green represent outliers. Each dot may represent multiple neurons.

Thirty-nine rats were used to study the feasibility, longevity, and quality of single units or field potentials captured by our novel stationary probes. Quantifiable results are presented in Table 3 and compared with other custom and commercial electrophysiology devices used for single unit recording in rodents. Single unit data was recorded in nine animals implanted with 32–64 channels targeting ACC (*N* = 2), OFC (*N* = 6), or both (*N* = 1). Histological analysis completed for 6/9 rats (ACC *N* = 1; OFC *N* = 5) confirms the location of our electrodes in target brain regions (Figure 3). Evidence of the ACC track was seen +3.2 through 2.5 mm AP; 0.6 mm ML; and reached 3.0 mm DV relative to bregma. The probe locations in OFC were seen from +4.0 through 2.5 mm AP; 2.0–2.8 mm ML; and 4.5–5.0 mm DV relative to bregma. In some cases, individual wires were seen to deflect up to ~150 μm (Figure 3). We were able to collect spikes with clean waveforms in both ACC and OFC (Figures 5A–D). In addition to meeting the criteria of having a low ISI violation (1.5% <3 ms), most single units we collected had a visible peak in the ISI histogram and produced separate clusters (k-mean) in space. We collected a total of 649 units across 104 behavioral sessions. The average yield (neurons/ brain region) was 7.32 ± 0.06 SEM in ACC and 5.81 ± 0.03 SEM in OFC (average of all sessions) (Figure 5E). The efficiency (neurons/ electrode) of our ACC probes were 0.23 ± 0.01 SEM while efficiency of OFC probes was 0.15 ± 0.01 SEM per session (Figure 5F). Our stationary probe was viable up to 24 weeks (168 days). The average longevity of all probes was 8 weeks (56 days). All animals with <5 weeks of data collection (*N* = 4) were ended early due to COVID-related lab shutdown and may not accurately reflect the chronic capabilities of our stationary probe design. The average cluster count (total number of neurons per session for each rat) peaked around 3–4 sessions (~2–4 weeks post-implantation), but then remained stable for the remainder of recording sessions (Figure 6A). The ACC neurons collected across all animals and sessions had an average firing rate of 4.8 ± 4.6 Hz (standard deviation), while OFC neurons had an average firing rate of 4.2 ± 4.7 Hz (standard deviation), consistent with previous literature (88–93). The average SNR, evaluated only for OFC neurons, was 11.74 ± 0.21 SEM across all sessions. The SNR holds steady across time; 11.85 ± 0.58 SEM on the first session of recording (*N* = 7 rats), 11.13 ± 0.72 SEM on the fourth session (*N* = 7 rats) and 12.86 ± 0.26 SEM after 11 sessions (*N* = 2 rats) (Figure 6B). Moreover, individual examples also show that the quality of our units generally does not deteriorate over time. Figure 6C shows example waveforms, spike counts, and ISI histogram of two neurons collected from the same polytrode captured 10 weeks apart. We did not implement measures to track neurons over multiple days and therefore cannot make claims about if these two units are the same neuron held over 10 weeks. Spiking activity was time-locked to behavioral events (trial onset or response) during the visual discrimination go/wait task. The average firing rate (baseline normalized) of all neurons during a “go” trial show that generally ACC and OFC neurons respond differently to the behavioral task (Figure 6D). ACC is more active for stimulus-mapping during the pre-response period and OFC is more active during outcome/reward evaluation. The waveform characteristics (hyperpolarization duration and peak-to-trough width) were analyzed with k-means clustering to visual putative amounts of excitatory/inhibitory neurons collected. Fifty-eight OFC neurons (15%) were classified as inhibitory, fast-spiking neurons (short-hyperpolarization, and small width) and 306 neurons (81%) were excitatory (longer-hyperpolarization and larger width) (Figure 6E). In the ACC neuronal cohort, 46 neurons (21%) were classified as inhibitory and 170 neurons (78%) as excitatory (Figure 6E) (94, 95), demonstrating the similarity of units found in both brain regions.

**Figure 7:**
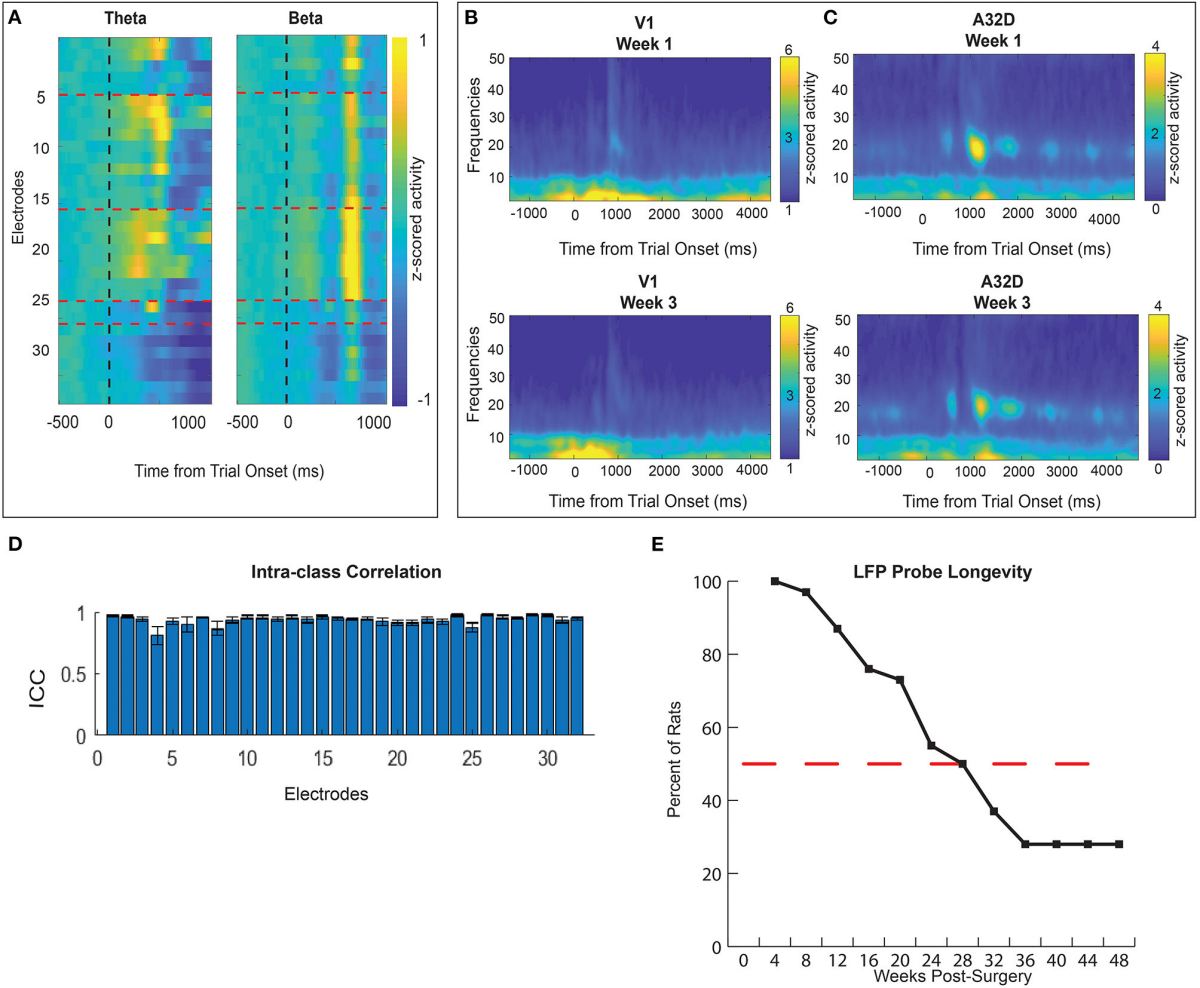
Quality of LFP signal displayed as time-frequency (TF) plots **(A–C)**. **(A)** A whole-brain map of time-locked activity at theta and beta frequencies across all 32 electrodes on “go” trials of the visual discrimination task. Normalized activity shows unique electrode x frequency patterns across time. Namely, electrodes 14–22 show the strongest reward related beta activity (13–30 Hz). Theta activity (4–8 Hz) in clusters of electrodes occurs earlier during response action. **(B,C)** TF activity from two example brain regions: visual cortex (V1) **(B)** and dorsomedial prefrontal cortex (A32D) **(C)**. “Go” trial normalized activity is time-locked to trial onset and displayed across frequencies for one animal's sessions. Activity is compared in one animal from two sessions recorded 2 weeks apart. **(D)** An intra-class correlation is used to assess how reliable data was from the same electrode across time (*N* = 61 sessions in *N* = 10 animals). An ICC score close to 1 indicates high similarity for that group of data. ICC scores were generated for all 32 electrodes based on data averaged across all (*N* = 10) rats. **(E)** LFP probe longevity is illustrated as the percent of rats with usable implants 1–48 weeks post-surgery. All 30 rats generated quality LFP signal 4 weeks post- surgery. The red dashed line marks the 50% point where at 28 weeks (7 months) 10 rats (out of 20 being recorded) still had usable physiology data. Four rats maintained quality signal until 48 weeks (12 months) post-surgery.

Our novel LFP probes were designed to measure distributed brain activity simultaneously from 32 different brain regions. Field potentials were evaluated from 30 animals. Histology was completed on 10/30 rats with LFP probes and generally confirms that we recorded from target brain areas (Figure 4). We first show that distinct patterns of activity can be measured from these different brain regions during an operant task (visual discrimination go/wait task). We found clearly distinct spectro-temporal patterns of activation in different brain regions, demonstrating that our probe design was able to minimize contamination of signals from different electrodes to capture activity from multiple brain regions at different frequencies (Figure 7). A whole-brain map of time-locked activity across all 32 electrodes illustrates how this “large-scale” brain-mapping approach can provide an unbiased sampling of brain-wide activity during the task (averaged across 10 animals, 61 sessions). Comparing activity in two distinct frequency bands illustrates how there are different distributions of activity at different time-points/frequencies (Figure 7A). A full account of the relationship between brain activity and behavior on this task has been published recently and will not be described here (79). One of our major goals was to develop a method for measuring brain activity that would be stable over time, thus allowing for a more sensitive way of measuring brain changes following injury or chronic manipulation. Although we previously examined spatio-temporal patterns time-locked to behavioral events (79), here we extend our investigation and further prove the utility of our probe design by looking at signal stability over a chronic period. We found a fair degree of stability in brain activity over time. For example (Figures 7B,C), we found that time-frequency activity from the same animal was fairly similar across a 2 week span in both areas. To quantify this, we calculated the intraclass-correlation (ICC) for each electrode across all sessions (*N* = 61) in 10 animals. We found a generally high ICC (Figure 7D) across sessions, clearly demonstrating the stability of our signal and utility of our probes for measuring longer-term changes associated with injury or learning; or to test out other interventions (such a pharmacology). Finally, we wanted to explore the longevity of these neural probes. Probes could fail for numerous reasons including head-stage becoming unattached from the animal; damage to the ground/reference wire connections on the EIB board; destruction to the omnetics connector. Across all animals (pooling from several studies, *n* = 30), our probes lasted from 5 to 56 weeks. We had good recordings from at least 50% of animals with implants 7 months after injury (Figure 7E). All 30 implants had good signal for at least 4 weeks and five implants were collecting viable signals at 48 weeks (12 months) post-implantation. One rat's implant lasted 56 weeks (14 months) until the study was concluded. Histological analyses confirm the location of our electrodes in target brain regions (Figure 4). In general, there was more variability in location and trajectory among deeper, more ventral wires. Our examples demonstrate the yield, longevity, and quality of our physiological data collected from our novel whole-brain LFP probe design.

## Discussion

To bridge our understanding between basic and clinical research it is necessary for even non-physiologists to procure affordable and scalable ways of measuring *in vivo* brain activity during translationally relevant behaviors. For true translational relevance, a behavior should not only have similar behavioral characteristics, it should also depend on similar electrophysiological circuits. Novel designs for physiology recording devices aim to increase their efficiency (quality, quantity, and longevity) while offering flexible designs that can be used for a multitude of experiments. Probes such as silicon microarrays, have attempted to improve collection efficiency by increasing the number of channels available for collection (31, 96, 97), while probes with thin wires or biocompatible insulation coating were designed in order to increase the longevity of recordings (72, 98). There are several challenges making stable extracellular signals from awake, behaving animals difficult to sustain over a long time period. Variability in probe success may depend largely on the induced trauma during surgical implantation and complicated inflammatory responses to the foreign body (99, 100). The uncertainty of extracellular recordings makes it particularly frustrating when large amounts of money and time are spent on devices that give poor neuronal yield or longevity. Attempting to simplify the probe fabrication and make physiology data collection more “user-friendly,” we sought to offer a simple, low-cost, time-efficient, solution to record chronically in rats performing operant tasks. Our design to collect single units is affordable (~$150), takes only 4–6 h to build, and is easily customizable to reach target brain areas with up to 64 channels (Table 3). Moreover, the probes are small and lightweight, critical for long-term studies in rodents. The LFP probe described offers a novel solution to record “brain-wide” activity simultaneously to achieve an unbiased sampling of physiological activity and are also low-cost, time-efficient solutions. Ultimately, information from both single units and field potentials can be used together to elucidate information about neurophysiology, relating micro and macro levels of network activity during cognitive behaviors.

Our low-cost, multi-site, stationary single unit probe includes 32–64 channels which is standard with most custom built fixed or brush microwires (43, 44, 59–63) but is limited compared to commercial probes with dense, “grid-like” arrays (45, 49, 50) and silicon Neuropixels probes (46–48). The cost of our probe is more affordable than commercial microdrives (51, 55, 64), shuttle drives (65), floating arrays (66, 67) and significantly less expensive than UTAH arrays (45, 49, 50) or Neuropixels (46) (Table 3). There are many commercial products that can be harnessed (as we have done) or replicated, to exponentially lower the cost of electrophysiology arrays, but performance may be compromised. Indeed, the performance of our probe assessed primarily by yield (neurons/area), efficiency (neurons/ recording site), and signal-to-noise ratio, was not as satisfactory as many commercial probes (Table 3). Several studies report yields >10 neurons/area where, on average, we report 5.82 neurons in OFC during a single recording session and 7.32 in ACC. Generally, a high yield is observed in the first few weeks that gradually diminishes over time, making it difficult to compare between experimental designs of different lengths (42, 101–103). Likewise, our efficiency (M = 0.15 in OFC and M = 0.23 in ACC) was less than other studies including similar custom fixed microwire arrays (61), but our average SNR of 11.74 was comparable to even commercial probes with high yields (47, 50, 63). Direct comparisons of efficiencies across studies is complicated due to differences in spike sorting methods and criteria. For instance, our probe is not built like a tetrode/polytrode with fixed distances between recording sites, but we utilized polytrode sorting as a conservative metric to compare spikes on 16 channel bundles. Therefore, it is most appropriate to compare our results with other fixed microwire and brush arrays, but these are typically sorted by individual electrodes which may inflate cell counts if neurons are detected at numerous recording sites (44, 60–63, 82, 83, 99). We captured stable spiking activities up to 24 weeks (168 days) post-implantation (M,= 56 days). Our probe's longevity was similar to other stationary probes recording single units from motor cortex, auditory cortex, hippocampus, and thalamus for ~40–80 days (44, 62, 99) (Table 3). Although it is difficult to assess the degree of brain damage due to surgical implantation, the low stiffness of our thin nichrome wires could theoretically reduce shearing and shear-related inflammation of adjacent brain tissues, giving our probes extended longevity necessary for chronic behavioral measures (31, 104). We did not assess if the same neuron could be held across multiple days, which is critical information for circuit analysis. We did observe waveforms that were consistent in shape and properties across multiple days but cannot make any assertions about whether this was the same neuron. Together, our stationary single unit probe is a satisfactory method for collecting chronic data, especially considering its low-cost, time-efficient and simplistic design compared to other studies.

We further demonstrated how our stationary probe design could be used to collect field potentials, an approach which may serve as a useful link between micro and macro- level circuits when used in conjunction with single unit recordings. The major advantages of our whole-brain LFP recording approach are: (1) The electrode recordings are fixed and remain stable for an average of 7 months (~200 days) and up to 14 months (>400 days), allowing researchers to study changes in activity after both short (i.e., pharmacological) or long-term (i.e., brain injury) manipulations. There are limited reports to date of *in vivo* physiology devices capturing quality signals across this length of time. (2) LFP can be used to capture rapid temporal dynamics, like our single unit probes, thus useful for measuring physiological activity during a precisely timed behavioral task. The versatility of our probe design makes it a useful tool for many behavioral applications and easy for even non-physiologists to implement. (3) Our probes are designed to measure activity from widespread brain areas across networks simultaneously, allowing for an unbiased view of physiology which is critical for brain mapping and network investigation. The design of having one cannula house four wires each cut to different lengths allows us to target many brain regions simultaneously while keeping surgical implantation as non-invasive as possible. This design is also custom in that a researcher is only limited by targeting brain regions with the same AP/ML coordinates and is scalable up to 64 channels. Additionally, the configuration can be adjusted to sample data from both hemispheres concurrently. França et al. (30) built a similar stationary probe to record field potentials and multi-unit activity in rodents also compatible with the Intan/Open-ephys system. Their design features a 16–32 channel array of 35–50 μm tungsten wires that is also time and cost-efficient to make (3 h; $100). The researchers were able to record LFP for up to 3 months (~90 days) in cortical and hippocampal sites. Our probes on average lasted 7 months and therefore may be beneficial for long-term studies. Moreover, the major difference in the two probes is the arrangement of electrode recording sites. França et al. (30) harness a “grid-like” design (3 × 10 with 250 μm between wires), facilitating the ease of fabrication, but limiting simultaneously accessible recording sites to those in close proximity. An advantage of our probe therefore is that all 8 cannula are built independently and can target different AP/ML coordinates, providing the ability to measure widespread signals across the brain.

### Limitations and Future Considerations

One disadvantage of our design is its inflexibility after implantation. Unlike with microdrives, there is no ability to move electrode sites vertically. The collection of spiking activity and field potentials may be affected by acute injuries of neural tissue, including inflammation, severe bleeding and chance of implanting in a “dead zone” (54, 73, 99). The histopathology does confirm damage around the electrode tracks after being implanted chronically for months (Figure 4). There was a tendency for cannula bundles with electrodes positioned closer together to produce more damage (Ex. Cannula 6 Figure 4). As other studies have suggested, coating the wires with bioactive materials, such as nerve growth factors, dexamethasone, and laminin, could alleviate injury response and promote neural regeneration (101, 105–107). New technologies such as commercially available silicon and polyimide probes aim to reduce the electrode's size and rigidity causing less inflammation and damage overtime and increasing the longevity and quality of signal, but these commercially available probes come at a cost (31, 34, 97, 106).

Our probes also show increased variability in performance at more ventral recording sites. Single unit ACC probes had better yield and longevity than electrodes in OFC. Deflection of wires (estimated up to 150 μm) in the brush-like formation was also observed more often in OFC arrays. Ventral sites targeted with our 32-CH LFP probe like nucleus accumbens, basolateral amygdala, and subthalamic nucleus, were more inconsistent and difficult to validate with our histological methods compared to dorsal sites (Figure 4, Table 2). In order to achieve “brain-wide” simultaneous recording, we restricted the number of electrodes per target area (usually 1 per area), and thus limit our potential to collect data from a brain site. These problems are exacerbated by the inherent difficulty to interpret field potentials as their contributions may come from multiple sources and be affected by volume conduction (34, 35, 41). One approach we could have taken (and one that others may use in the future) is to reduce the number of sites we target by half and use bipolar electrodes as a local reference for any electrode of interest. However, local referencing itself requires various choices to be made (e.g., the distance between electrode sites will affect what is “referenced out”), and across both superficial and deep targets it's unclear how to ensure a consistent spacing of such electrodes. Given these complexities, we have ended up using a median referencing approach, followed by comparison of activation profiles across brain regions. While this limits the specific localization, it does allow for broad generalization to be made (as is evidenced by the data above), in which certain regions clearly show more or less activity, which can provide support for where activity may be arising. Finally, in recent analyses we have utilized methods of functional connectivity like weighted phase-lagged index [see (79)], which inherently suppress volume conduction artifacts, allowing us to better demonstrate that activity within and between regions are not simply a result of volume conduction (which would have strong 0-phase-lagged relationships).

Although spikes are easier to interpret, there is a general concern of sufficient spike sorting with high-density probes, a problem that is more apparent with commercially available probes exceeding hundreds of recording sites (41, 73, 84, 86). With extremely high-density probes (such as Neuropixels), sorting occurs automatically, using data from all channels simultaneously (48, 108). Even with a simple multi-channel electrode design like ours, not only does one electrode detect multiple neurons, but the same neuron may be detected on multiple electrodes. To address this problem, we sorted in bundles of 16 wires (two fixed cannula) as opposed to individual channels to mitigate the potential overlap in spike detection from our close electrode sites. Polytrode or tetrode probes are constructed with recording sites at fixed distances to capture multiple views of the same neuron, more beneficial for spike sorting (54, 73, 80, 81, 84–87). Our probe is not designed like a polytrode/tetrode and therefore still suffers from all the normal limitations of a standard microwire array. At “worst” case, all electrodes contained completely unique information (i.e., were far enough from each other as to not contain any shared/common information). Thus, at “worst” case, we would be able to sort as well as micro-wires, while at “best case,” sorting of neurons would be improved if some of the 16 electrodes implanted together sampled common activity from the same neuron (which was observed in our data; Figure 5). We recognize that this may be a limitation to our results.

Here, we demonstrate how stationary probes designed to capture single units and field potentials can be used to investigate physiological signals associated with cognitive behaviors. Our designs offer unique advantages over other electrophysiology recordings devices in terms of offering a simple, affordable and customizable approach to study physiology data over a long period of time. Our aim in designing fixed, low-cost probes is to offer a simple method of collecting physiology that can complement behavioral data from animal models to inform clinical research on neurobiological and psychiatric disorders.

## Data Availability Statement

The raw data supporting the conclusions of this article will be made available by the authors, without undue reservation.

## Ethics Statement

The animal study was reviewed and approved by San Diego VA Medical Center Institutional Animal Care and Use Committee.

## Author Contributions

MF, TT, NB, and DR designed single unit probes and conducted experiments. MF, LF, NB, SH, and DR designed field potential probes and conducted experiments. MF, TT, LF, XW, SH, JC, and DR analyzed data. MF, TT, JC, and DR wrote paper. All authors contributed to the article and approved the submitted version.

## Conflict of Interest

The authors declare that the research was conducted in the absence of any commercial or financial relationships that could be construed as a potential conflict of interest.

## Publisher's Note

All claims expressed in this article are solely those of the authors and do not necessarily represent those of their affiliated organizations, or those of the publisher, the editors and the reviewers. Any product that may be evaluated in this article, or claim that may be made by its manufacturer, is not guaranteed or endorsed by the publisher.
